# The National Audit of Primary Biliary Cholangitis (PBC) in the United Kingdom: Defining the Audit Dataset and Data Collection System

**DOI:** 10.7759/cureus.25609

**Published:** 2022-06-02

**Authors:** Laura Burke, Steve Flack, Rebecca Jones, Richard J Aspinall, Douglas Thorburn, David E Jones, Conor Braniff, Collette Thain, Andrew Yeoman, Joanna A Leithead, Palak J Trivedi, George Mells, Laith Alrubaiy

**Affiliations:** 1 Hepatology, Leeds Teaching Hospitals Trust, Leeds, GBR; 2 Medical Genetics, University of Cambridge, Cambridge, GBR; 3 Hepatology, Leeds Liver Unit, St. James's University Hospital, Leeds, GBR; 4 Gastroenterology and Hepatology, Portsmouth Liver Centre, Queen Alexandra Hospital, Portsmouth, GBR; 5 Hepatology, Royal Free Hospital, London, GBR; 6 Hepatology, Centre for Liver Research, Newcastle University, Newcastle upon Tyne, GBR; 7 Hepatology, Regional Liver Unit, Belfast Health and Social Care Trust, Belfast, GBR; 8 Chief Executive Officer, The PBC Foundation, Edinburgh, GBR; 9 Hepatology, Gwent Liver Unit, Royal Gwent Hospital, Newport, GBR; 10 Hepatology, Forth Valley Royal Hospital, Larbert, GBR; 11 Hepatology, University Hospitals Birmingham NHS Foundation Trust, Queen Elizabeth Hospital, Birmingham, GBR; 12 Gastroenterology, St. Mark's Hospital, London, GBR

**Keywords:** health service research, hepatology, quality improvement research, audit, primary biliary cholangitis

## Abstract

Primary biliary cholangitis (PBC) is a debilitating chronic liver disease that progresses to cirrhosis with attendant complications in a substantial proportion of patients. It is a major cause of liver-related morbidity and mortality in the United Kingdom (UK). The British Society of Gastroenterology (BSG) published guidelines on PBC management, which included key audit standards. Therefore, we propose the first UK-wide audit of the management of PBC, sanctioned by the BSG and the British Association for Study of the Liver (BASL), to benchmark NHS trusts and health boards against these audit standards as a guide to targeted improvement in the delivery of PBC-related health care.

## Introduction

Primary biliary cholangitis (PBC) is a chronic, cholestatic liver disease characterized by autoimmune destruction of the small, intrahepatic bile ducts, causing chronic cholestasis and progressive fibrosis, culminating in biliary cirrhosis [1]. The prevalence rate of PBC in the United Kingdom (UK) is estimated to be 25 per 100,000 of the total population, suggesting there are approximately 17,000 PBC patients in the United Kingdom [2].

Primary biliary cholangitis is most often diagnosed in women over the age of 50 years (Female:Male {F:M} = 10:1) [3]. Typical symptoms include pruritus, fatigue, memory disturbance (brain fog), and dry eyes and mouth. Other features include hyperlipidemia and osteoporosis. Complications of PBC-related cirrhosis, as in other forms of cirrhosis, include portal hypertension, chronic liver failure, and hepatocellular carcinoma (HCC). PBC is associated with other autoimmune conditions, such as Sjögren syndrome, systemic sclerosis, and systemic lupus erythematosus [4]. Clinical manifestations are highly variable, however, rates of asymptomatic disease have increased in recent years, owing in part to routine testing of liver biochemistry [3].

Updated guidelines for managing patients with PBC were published in 2018 by the British Society of Gastroenterology (BSG) in collaboration with UK-PBC [1]. These summarize current evidence for the diagnosis and management of PBC. The key elements of these guidelines are as follows: the diagnosis of PBC is confirmed by two of the following criteria - (1) persistent, unexplained cholestatic liver biochemistry; (2) detection in the serum of anti-mitochondrial antibodies (AMA) or the PBC-specific anti-nuclear antibodies (ANA), anti-Sp100 and anti-gp210 antibodies; or (3) liver biopsy features compatible with PBC [1]. However, liver biopsy is not recommended except where there is suspicion of seronegative PBC, or PBC with features of autoimmune hepatitis (AIH), also known as PBC-AIH overlap syndrome [1].

Treatment recommendations include optimizing the dose of first-line therapy, ursodeoxycholic acid (UDCA), to 13-15 mg/kg/day; risk-stratify patients based on UDCA response to determine their suitability for second-line therapy (see below); regularly evaluating symptoms; regularly assess the risk of osteoporotic fracture; surveil patients with cirrhosis for HCC and gastroesophageal varices (GOV); and consider liver transplantation (LT) for patients with a serum total bilirubin ≥50 mmol/L or UKELD score ≥49 [1,5].

Patients with PBC may be stratified into low or high-risk groups based on their liver biochemistry on treatment with an optimal dose of UDCA, so-called "UDCA response." There are many definitions of UDCA response, the industry standard being a serum alkaline phosphatase (ALP) <1.67 times the upper limit of normal (ULN) after ≥12 months of treatment with UDCA 13-15 mg/kg/day [6]. Approximately 30% of PBC are UDCA non-responders, with ALP ≥1.67×ULN despite treatment with UDCA [7]. These patients have a substantially increased risk of disease progression [8]. Therefore, UDCA non-responders (and patients who are intolerant of UDCA) should be considered for second-line therapy with obeticholic acid (OCA). In the United Kingdom, UDCA non-responders may also be offered second-line therapy with a fibrate, bezafibrate, or fenofibrate, although fibrates are presently not licensed for this indication.

Following the publication of the BSG guidelines, we conducted a pilot audit of the management of PBC in 11 National Health Service (NHS) trusts or health boards in England, Wales, and Scotland [9]. We reviewed data from 790 patients with PBC and found that most participating centers had not fulfilled all the recommended standards. Notably, we found significant variation in optimal prescribing of UDCA; risk stratification using the UDCA response; risk assessment for osteoporotic fracture; assessment of the symptoms, pruritus and fatigue; and referral of high-risk patients for LT [9].

These figures imply that management of PBC in the United Kingdom might be sub-optimal. Therefore, we propose a UK-wide audit, supported by national bodies, to evaluate the management of PBC across the United Kingdom as a springboard to targeted improvement in PBC-related health care, especially in relation to the provision of second-line therapy.

We planned a nationwide audit to determine whether NHS liver centers have fulfilled the audit standards proposed in the BSG guidelines, identify discrepancies in the provision of PBC-related health care across the United Kingdom, and identify specific deficiencies for targeted improvement. Specifically, the audit will benchmark clinical practice against the standards published by the BSG [1]. This study aimed to define the dataset for the audit and the audit collection method to meet the audit objectives.

## Materials and methods

The project included discussions with hepatologists, patients, IT specialists, and clinicians who look after patients with PBC. We divided the discussions into several workstreams to develop the registry and meet the requirements. They include data collection workstreams to identify data needed to be collected for the audit that meet the national guidelines and support the patient's care; technical IT infrastructure workstream to develop the IT software needed for the audit, this has to be user friendly and compatible with ethics committee requirements; steering committee workstream to oversee the work of the audit and ensure all clinical governance aspects are met.

## Results

We planned a UK-wide, retrospective audit on the management of PBC patients. The audit is sanctioned by the BSG and the British Association for the Study of the Liver (BASL). Therefore, all NHS liver units will participate; other NHS trusts and health boards that provide liver services but are not considered liver units will be strongly encouraged to participate. The audit will be registered with the audit office of each participating center, and each participating center will nominate a local audit lead and audit team. We anticipate that the local audit lead will be a consultant gastroenterologist or hepatologist, and the local audit team will include a gastroenterology or hepatology trainee or specialist nurse. The audit will include all patients with a confirmed diagnosis of PBC or PBC-AIH overlap syndrome, who are under follow-up at the participating center at the time of data collection.

Robust case-finding strategies will be employed through interrogation of clinical coding databases for all patients with an inpatient or outpatient International Classification of Diseases (ICD)-10 code for PBC or PBC-AIH overlap syndrome, interrogation of immunology laboratory databases for patients with a positive test for AMA or PBC-specific ANA, interrogation of histopathology laboratory databases for patients with a histological diagnosis of PBC or PBC-AIH overlap syndrome, interrogation of gastroenterology or hepatology departmental databases for patients with PBC or PBC-AIH overlap syndrome. Furthermore, centers collaborating in UK-PBC can be provided with an up-to-date list of PBC patients recruited into the UK-PBC Research Cohort at that center.

We anticipate that case-finding might identify individuals who fulfill the diagnostic criteria for PBC but have not been referred to the local gastroenterology or hepatology clinic. Though important, non-referral of PBC patients to secondary care is not the topic of this audit. We expect that where PBC patients are identified who are not under specialist follow-up, the relevant audit team will write to the patient’s general practitioner (GP) to recommend referral. 

Data collection

The local audit team will collect retrospective patient data from hospital medical records. We anticipate that these data will be available from electronic medical records in most cases. Data collection will be completed using REDCap (Research Electronic Data Capture; Nashville, TN: Vanderbilt University), a secure web-based data collection tool licensed for the University of Cambridge UK-PBC Cohort. On agreeing to participate in the audit, centers will be provided with the audit form. The data collected for the audit were defined by the steering committee (Table 1). Optional data can be added such as fibroscan results, and associated autoimmune diseases such as thyroiditis, and sicca syndrome. Patients will be identifiable locally, but no patient identifiers will be included in the national aggregation of data for analysis. For the avoidance of doubt, data will be stripped of any patient identifiable information before submission to the UK-PBC data manager.

**Table 1:** Data to be collected for the United Kingdom national audit. OCA: obeticholic acid; HCC: hepatocellular carcinoma; GOV: gastroesophageal varices; FRAX: Fracture Risk Assessment Tool; DEXA: dual-energy x-ray absorptiometry; BDM: bone mineral density; SLT: second-line therapy; UDCA: ursodeoxycholic acid

Treatment center
Patient age, sex, and weight
Results of contemporaneous laboratory investigations, including liver biochemistry, renal biochemistry, full blood count and blood clotting
Use of UDCA and dose, or documented reason for non-use of UDCA, where applicable
Record of response to UDCA
Record of risk stratification
Referral of UDCA non-responders for SLT, where applicable, or documented reason for non-referral of UDCA non-responder for SLT, where applicable
Use of OCA and dose, where applicable
Use of fibrates and dose, where applicable
Record of assessment for pruritus and fatigue
Use of specific anti-pruritic treatment (e.g. cholestyramine or rifampicin), where applicable
Record of BMD assessment (FRAX score or DEXA scan) within the last five years, and actions taken, if applicable
Record of HCC surveillance, where applicable
Record of GOV screening, where applicable
Record liver biopsy, where applicable
Record of referral to a liver transplant center, where applicable

After local data collection is complete, the local audit team will identify and remove duplicates; strip the data of patient identifiable information, and submit the anonymized data to the UK-PBC data manager. The Data Manager will perform quality control (QC) checks. He will liaise with local audit teams to resolve any omissions or discrepancies in the data. Once all datasets have been reviewed and approved, the data will be merged into a single master database.

Averages and percentages for each standard will be calculated for individual sites and compared. Statistical analysis will be undertaken to identify areas of significant difference. Consideration will be given to sites with few PBC patients (under 20), where percentages are likely to provide an exaggerated presentation.

Ethics and governance

The audit is a service evaluation tool, no identifiable patient information will be shared, and the management of individual patients will not be affected. However, the audit will be registered with each center’s audit office prior to data collection in that center. NHS code of confidentiality will be followed at all times.

Dissemination

Results from the audit will be shared with participating centers; presented at the local, national, and international meetings. Audit results will be discussed with national societies, patients’ representatives, and regulatory bodies to identify areas for improvement and maximize patient care.

## Discussion

While PBC is an uncommon disease, it can significantly impact patients’ daily lives and has a substantial demand on NHS services. Updated guidelines for managing patients with PBC were published in 2018 by the British Society of Gastroenterology (BSG) in collaboration with UK-PBC [1]. A recent study showed that the care for PBC had not fulfilled all the recommended standards [9]. Of concern, the study found significant geographical variation in optimal prescribing of UDCA; risk stratification using the UDCA response; risk assessment for osteoporotic fracture; assessment of the symptoms, pruritus, and fatigue; and referral of high-risk patients for LT [9]. The European Association for the Study of the Liver (EASL) published similar guidelines to standardize the management of PBC and improve care [10].

Quality of life and symptomatic improvement play a large role in PBC management. Patients with early-onset PBC are reported to have a lower quality of life and have experienced more severe progressive disease. Therefore, monitoring symptoms and quality of life alongside biochemical results are crucial to the management of this disease. With the development of recent screening tools such as the PBC-10, this can be addressed by clinicians [11].

UDCA treatment has represented the standard of care for decades. However, with the emergence of new treatment options, clinicians need to ensure that their care is up-to-date with existing guidelines to improve quality of life, prevent disease progression, and identify and direct non-responders to second-line therapies.

Establishing a project steering committee and involving all stakeholders, this project defined the dataset and IT software to support the first national audit in the United Kingdom to identify inconsistencies in PBC management in the United Kingdom and translate the findings into the development of a PBC care pathway to improve the quality of care and patient outcomes.

## Conclusions

This project helped to set up the first national registry for patients with PBC. The audit will identify gaps in the care for patients with PBC and will be used as a quality improvement tool to improve the care of patients. Future data collection will show its effectiveness and usefulness.

## References

[1] Hirschfield GM, Dyson JK, Alexander GJ (2018). The British Society of Gastroenterology/UK-PBC primary biliary cholangitis treatment and management guidelines. Gut.

[2] James OF, Bhopal R, Howel D, Gray J, Burt AD, Metcalf JV (1999). Primary biliary cirrhosis once rare, now common in the United Kingdom?. Hepatology.

[3] Selmi C, Bowlus CL, Gershwin ME, Coppel RL (2011). Primary biliary cirrhosis. Lancet.

[4] Gershwin ME, Selmi C, Worman HJ (2005). Risk factors and comorbidities in primary biliary cirrhosis: a controlled interview-based study of 1032 patients. Hepatology.

[5] Barber K, Madden S, Allen J, Collett D, Neuberger J, Gimson A (2011). Elective liver transplant list mortality: development of a United Kingdom end-stage liver disease score. Transplantation.

[6] Nevens F, Andreone P, Mazzella G (2016). A placebo-controlled trial of obeticholic acid in primary biliary cholangitis. N Engl J Med.

[7] Carbone M, Nardi A, Flack S (2018). Pretreatment prediction of response to ursodeoxycholic acid in primary biliary cholangitis: development and validation of the UDCA response score. Lancet Gastroenterol Hepatol.

[8] Carbone M, Sharp SJ, Flack S (2016). The UK-PBC risk scores: derivation and validation of a scoring system for long-term prediction of end-stage liver disease in primary biliary cholangitis. Hepatology.

[9] Sivakumar M, Gandhi A, Shakweh E (2022). Widespread gaps in the quality of care for primary biliary cholangitis in UK. Frontline Gastroenterol.

[10] (2017). EASL Clinical Practice Guidelines: the diagnosis and management of patients with primary biliary cholangitis. J Hepatol.

[11] Alrubaiy L, Mells G, Flack S, Bosomworth H, Hutchings H, Williams J, Jone D (2019). PBC-10: a short quality of life measure for clinical screening in primary biliary cholangitis. Aliment Pharmacol Ther.

